# Trace Elements Levels in Major Depressive Disorder—Evaluation of Potential Threats and Possible Therapeutic Approaches

**DOI:** 10.3390/ijms242015071

**Published:** 2023-10-11

**Authors:** Jacek Baj, Julia Bargieł, Justyna Cabaj, Bartosz Skierkowski, Gabriela Hunek, Piero Portincasa, Jolanta Flieger, Agata Smoleń

**Affiliations:** 1 Department of Anatomy, Medical University of Lublin, Jaczewskiego 4, 20-090 Lublin, Poland; 2 Student Research Group of Department of Epidemiology and Clinical Research Methodology, Medical University of Lublin, Radziwiłłowska 11, 20-080 Lublin, Poland; juliabargiel@interia.pl (J.B.); justyna.99@vp.pl (J.C.); skierabartek@gmail.com (B.S.); 3 Student Research Group of Department of Forensic Medicine, Medical University of Lublin, Jaczewskiego 8b, 20-090 Lublin, Poland; hunekgabriela@gmail.com; 4 Clinica Medica “A. Murri”, Department of Biomedical Sciences & Human Oncology, University of Bari Medical School, 70124 Bari, Italy; piero.portincasa@uniba.it; 5 Department of Analytical Chemistry, Medical University of Lublin, Chodźki 4A, 20-093 Lublin, Poland; jolanta.flieger@umlub.pl; 6 Department of Epidemiology and Clinical Research Methodology, Medical University of Lublin, 20-080 Lublin, Poland; agata.smolen@umlub.pl

**Keywords:** major depressive disorder, depression, trace element, psychiatry, metallomics

## Abstract

The multifactorial etiology of major depressive disorder (MDD) includes biological, environmental, genetic, and psychological aspects. Recently, there has been an increasing interest in metallomic studies in psychiatry, aiming to evaluate the role of chosen trace elements in the MDD etiology as well as the progression of symptoms. This narrative review aims to summarize the available literature on the relationship between the concentration of chosen elements in the serum of patients with MDD and the onset and progression of this psychiatric condition. The authors reviewed PubMed, Web of Science, and Scopus databases searching for elements that had been investigated so far and further evaluated them in this paper. Ultimately, 15 elements were evaluated, namely, zinc, magnesium, selenium, iron, copper, aluminium, cadmium, lead, mercury, arsenic, calcium, manganese, chromium, nickel, and phosphorus. The association between metallomic studies and psychiatry has been developing dynamically recently. According to the results of current research, metallomics might act as a potential screening tool for patients with MDD while at the same time providing an assessment of the severity of symptoms. Either deficiencies or excessive amounts of chosen elements might be associated with the progression of depressive symptoms or even the onset of the disease among people predisposed to MDD.

## 1. Introduction

Major depression is one of the most common mental health disorders in the world and is marked by ongoing feelings of sadness, despair, loss of energy, and difficulty dealing with normal daily life. An estimated 3.8% of the population experience depression, including 5% of adults (4% among men and 6% among women) and 5.7% of adults older than 60 years, according to the World Health Organization (WHO). Approximately 280 million people in the world have depression, which is the leading cause of disability worldwide [1,2,3]. Further, major depressive disorder (MDD) affects about 17.3 million American adults, and according to the Centers for Disease Control data, 1.9 million children aged 3–17 have been diagnosed with depression [4]. According to the National Institute of Mental Health, neuropsychiatric disorders constitute the leading cause of disability in the U.S., with depression being the most prevalent; in fact, depression is ranked as the leading cause of disability worldwide [4]. Moreover, the prevalence of depression is high in cases of the coexistence of other diseases such as cancer, cardiovascular diseases, or neurodegenerative diseases, including Alzheimer’s or Parkinson’s disease [5]. Regarding the costs that are generated by the depression in the U.S., those are estimated at about 187.8 billion dollars per year [6]. The concept of depression covers a wide range of various types of depressive disorders, occurring both in the form of isolated depressive episodes and in the course of various diseases: other mental diseases, endocrinopathies, neurological diseases, infectious diseases, and others (i.e., cancer, rheumatoid arthritis) [7,8,9,10,11,12,13,14,15,16,17]. There are several subtypes of depression, such as major depression, persistent depressive disorder, bipolar disorder, psychotic depression, seasonal affective disorder, ‘situational’ depression, peri/postpartum depression, or premenstrual dysphoric disorder [18]. The risk of major depression is both genetically and environmentally determined. The most important predisposing factors include genetic disorders, especially those related to neurotransmitters, neurodegenerative diseases, severe and traumatic life events, excessive consumption of alcohol, or chronic stress [19,20,21,22,23,24,25,26,27]. In addition, nutritional deficiencies, such as vitamin D, group B vitamins, protein, selenium, iron, calcium, zinc, magnesium, or Omega-3 fatty acids, might significantly contribute to the appearance of depressive symptoms [5]. With regards to the alterations in the levels of monoamine neurotransmitters and the occurrence of specific symptoms of depression, primarily dopamine, serotonin, and norepinephrine are involved [28]. Importantly, the amount of trace elements plays a key role in the proper functioning of the nervous system and, as a result, appropriate psychiatric conditions. Even small deficiencies can lead to clinically significant changes and the occurrence of disease symptoms (Table 1) [27,28,29,30,31,32,33,34,35,36].

In addition, trace element imbalances can be indicators of disease remission or progression. It has been proven that there is a correlation between the concentrations of trace elements such as copper, iron, chromium, zinc, selenium, and magnesium and the proper functioning of the nervous system. Even small deviations from normal values can induce neurological and psychiatric diseases such as Alzheimer’s, Parkinson’s, Huntington’s disease, and dementia, which exacerbate the symptoms of autism spectrum disorder (ASD) and attention deficit hyperactivity disorder (ADHD) [27,102,103,104,105,106,107,108,109,110,111]. There is a suspicion that disturbances in the normal concentrations of trace elements may have an impact on the induction or exacerbation of depressive symptoms. Nutritional deficiencies resulting in disturbances in the amount of trace elements can lead to drowsiness, chronic fatigue, and loss of appetite [112,113,114,115]. Furthermore, a relationship was also found between the role of trace elements and the occurrence of apathy and anhedonia [116,117].

## 2. Aim of the Study and Search Strategy

The aim of the study was to review the available literature on the concentrations of trace elements in the serum of patients with depression and then analyze the results in terms of potential therapeutic use as well as possible clinical implications. The PubMed, Web of Science, and Scopus databases were searched to assess the papers devoted to the connection between depression and the role of the trace elements with the appropriate details. Regarding the time frames of the search, we chose the publications from the earliest available articles up to July 2023. The first identification performed in July 2023 included the search strategy as follows: (depression OR depressive disorder) AND (trace element). After a selection of articles searching for serum trace element concentrations that had already been investigated in sera of patients with depression, we chose the following ones to include in this review: zinc, magnesium, selenium, iron, copper, aluminium, cadmium, lead, mercury, arsenic, calcium, manganese, chromium, nickel, molybdenum, phosphorus, uranium, and antimony. We continued as the second identification with the use of the following search string: (depression OR depressive disorder) AND (zinc OR magnesium OR selenium OR iron OR copper OR aluminium OR cadmium OR lead OR mercury OR arsenic OR calcium OR manganese OR chromium OR nickel OR molybdenum OR phosphorus OR uranium OR antimony). There were no restrictions regarding the year of publication. The authors chose only articles in English; non-English articles were excluded from this review. Regarding the publications chosen during the search and ultimately reviewed in our article, there were no restrictions regarding the age of patients or their gender. We chose articles that involved both human and animal subjects.

## 3. Results

Since a relationship between depression and metallomics has been one of the most popular research topics for many years, the total number of articles was over two hundred thousand. Despite the application of restrictions such as disqualification of duplicates, case reports, comments to other papers, letters to editors, and papers devoted to other topics and selecting only free full articles in English, the number of papers was still appalling. The articles reviewed in this paper were evaluated primarily in terms of their specific subject matter, the level of compliance with the principles of conducting epidemiological research, the year of publication of the article (from the latest, with the limitation of those issued 10 years ago), and the number of citations. Thus, the articles most relevant to the possible role of trace elements in the context of depression were selected.

## 4. Zinc

Zinc, as an essential trace element, plays a crucial role in various biochemical and physiological processes within the CNS, contributing to proper brain development and function [118,119]. Acting as a cofactor for over 300 enzymatic reactions, zinc participates in gene transcription, DNA repair, cell growth, neurogenesis, and protein synthesis [119,120]. Moreover, zinc is known to modulate immune and inflammatory processes, impacting inflammatory cytokine levels [119]. Notably, zinc has been identified as an antagonist of the NMDA receptor, leading to the downregulation of the Glu response [118,120,121]. This element’s proper levels are crucial in brain regions associated with depressive symptoms, including the cerebral cortex, hippocampus, and amygdala [118]. Zinc deficiency may lead to an increase in hypothalamic-pituitary-adrenal (HPA) axis activity, resulting in elevated glucocorticoids and subsequent hippocampal dysfunction and behavioral abnormalities [122]. This hyperactivation of the HPA axis can disrupt serotonergic and noradrenergic circuits, affecting mood regulation [122,123]. Regarding zinc’s role in neurotransmission, it is primarily responsible for the modulation of various postsynaptic receptors, including NMDA and AMPA/kainite Glu receptors; zinc is also crucial to potentiate glycine-mediated currents as well as to regulate voltage-gated calcium channels [124,125,126,127]. Current research indicates that zinc is highly involved in the functioning of the serotonergic system, presenting its anti-depressant-like properties, which have been observed in both preclinical and clinical studies [128]. Zinc, depending on its concentrations, might act either as an agonist or antagonist of 5-HT_1A_ receptors. Apart from the control of serotonin levels, zinc is also involved in the control of dopamine release via the regulation of dopamine transporters by Zn^2+^ [129].

Zinc deficiency has been significantly associated with MDD and depression symptom severity [119,130]. Except for MDD, other psychiatric/neurological conditions such as psychosis, Alzheimer’s disease, stroke, or epilepsy might appear because of the impaired levels of zinc in the human organism. Nevertheless, it should be noted that zinc supplementation should be very careful since excess zinc might lead to neurotoxicity [44].

Studies evaluating zinc supplementation in conjunction with antidepressant therapy in MDD treatment have shown promising results. An inverse correlation was found between zinc concentrations and depression severity, with higher zinc levels associated with better treatment outcomes [131]. Patients receiving selective serotonin reuptake inhibitors (SSRIs) and higher zinc intake exhibited a significant reduction in depressive symptoms compared to those with lower zinc intake [132]. A meta-analysis by Swardfager et al. showed a correlation between greater mean symptom severity in MDD patients and significant differences in zinc levels compared to controls [130]. Furthermore, patients with treatment-resistant depression exhibited lower zinc concentrations compared to treatment-non-resistant patients [119,121,131]. These findings indicate that zinc supplementation could serve as a beneficial adjunct to antidepressant treatments, and measuring zinc blood concentrations may potentially serve as a biological marker for monitoring MDD severity [133].

Several hypotheses have been proposed to explain how zinc may influence depression and its neural transmission. Zinc’s interaction with glutamatergic transmission in the CNS, limbic system, and cerebral cortex may be a critical factor in depression development [119]. Furthermore, zinc deficiency may lead to increased production of ROS and NMDA agonists, contributing to excitotoxicity and serotonin depletion [134]. ROS are associated with oxidative stress, which has been described as highly relevant in the etiology of MDD [135]. Zinc also counteracts neurotoxicity caused by chronic inflammation via the NFκB pathway, impacting pathways associated with MDD [136].

Zinc impact on neuropeptide Y (NPY) is another aspect that needs further evaluation. NPY, involved in various brain regions linked to MDD, regulates food intake, sleep, and the HPA axis [137,138,139]. Zinc deficiency may disrupt NPY release and regulation, potentially affecting physiological functions associated with depressive symptoms [138,140].

Zinc’s role as a neuromodulator and its interaction with the G-protein-coupled receptor ZnR/GPR39 have also drawn attention [141,142,143,144]. ZnR/GPR39 activation triggers biochemical pathways associated with cell proliferation, anti-apoptotic properties, and neuroplasticity, making it a potential therapeutic target for MDD [142].

Additionally, zinc’s function as a cofactor for histone deacetylases suggests it may be involved in chromatin dynamics and transcription, contributing to the molecular basis of MDD [145,146].

In conclusion, zinc’s involvement in various neural pathways and mechanisms related to depression underscores its potential as a therapeutic target for MDD. Zinc supplementation, combined with antidepressant therapy, has shown promise for improving depressive symptoms. However, it is essential to be cautious about zinc supplementation since excessive intake could lead to neurotoxicity. Further well-designed studies, especially in specific populations like children and adolescents, are necessary to establish the significance of zinc in preventing and treating depression [147]. This knowledge could lead to better strategies for addressing depression and improving patient outcomes.

## 5. Magnesium

Magnesium (Mg) is an essential trace element known to play a significant role in the etiology, progression, and treatment of depression [148]. As the body’s fourth most abundant cation and the second most significant intracellular cation, magnesium acts as a cofactor in over 350 enzymes, many of which are involved in brain function and mood regulation [148]. Studies in animals and humans have highlighted the beneficial effects of magnesium supplementation on depression, emphasizing its relevance to the limbic system and its potential role in the development and course of depression [118]. Disturbed levels of magnesium might lead to the occurrence of such disorders as MDD, schizophrenia, anxiety and eating disorders, ADHD, or ASD [149].

In addition to its role in mood regulation, magnesium is involved in various physiological processes, such as neuromuscular function, cardiovascular health, and electrolyte balance [53,150]. Magnesium deficiency may result from insufficient dietary intake, renal loss in certain diseases like diabetes and alcoholism, and the use of certain medications [151]. Given its importance in energy generation, dysrhythmia prevention, blood pressure stabilization, insulin resistance prevention, and bone homeostasis, magnesium supplementation emerges as a promising therapeutic approach for individuals with depression [152].

Recent research has indicated that adding magnesium supplements to SSRI treatment can significantly improve symptoms of MDD [153]. Further investigations are warranted to explore the association between dietary magnesium intake and depression risk, as well as the potential gender-specific differences in magnesium levels among individuals with depression [154,155]. Increased consumption of magnesium-rich foods, such as nuts, green vegetables, and whole grains, could be advocated as a preventive measure for depression [155].

The role of magnesium as a cofactor for numerous enzymes, its impact on CNS function, and its ability to antagonize NMDA Glu receptors underscore its relevance in depression management [118,156,157]. Magnesium deficiency can lead to NMDA receptor hyperactivity, contributing to the development of depressive and anxiety-like symptoms and increased inflammatory markers [158]. Both animal and human studies have reported the antidepressant effects of magnesium supplementation [159,160,161,162].

The multifaceted role of magnesium in the CNS and its connection to depression highlight its potential as a valuable therapeutic option for MDD. Magnesium supplementation, when combined with standard antidepressant treatment, may lead to significant improvements in depressive symptoms. However, caution should be exercised in determining the optimal dosage and considering individual needs, as excessive magnesium supplementation can lead to neurotoxicity and other adverse effects [133].

Magnesium’s involvement in various aspects of CNS function and its association with depression suggest its potential as a valuable therapeutic option for individuals with MDD. Supplementing magnesium in conjunction with standard antidepressant treatment may yield significant benefits in managing depressive symptoms. A quite recent randomized controlled trial demonstrated that magnesium supplementation might be effective for mild to moderate depression in adults, regardless of age, gender, or usage of antidepressant treatment [159]. The daily dosage of elemental magnesium used in the abovementioned study was 248 mg; such a dosage was well tolerated by the patients, and according to the scientists, it was safe enough at this dosage not to closely monitor its toxicity in patients. It was shown that supplementation of magnesium in dosages between 125 and 300 mg might be beneficial for patients with MDD, resulting in the alleviation of depressive symptoms [160]. Nevertheless, it should be noted that high doses of magnesium might be harmful; therefore, it would be beneficial to provide proper ranges of dosages that would help patients with MDD while at the same time not being harmful to the patient. Further research is warranted to determine the optimal dosage, long-term effects, and potential gender-specific response to magnesium therapy. Introducing magnesium-rich foods or magnesium supplements as part of depression management may pave the way for more effective and personalized treatment strategies for individuals with depressive disorders [133].

## 6. Selenium

Selenium is an indispensable component of the brain’s antioxidant system, primarily due to the activities of selenoproteins such as glutathione peroxidases (GPx) and thioredoxin reductases [163]. Additionally, selenium plays a role in regulating mitochondrial biogenesis and calcium channels, and experimental studies have indicated its association with increased brain-derived neurotrophic factor synthesis, which is crucial for neuroplasticity [163]. Biological functions of selenium in the CNS primarily manifest in the action of selenoproteins that are responsible for the proper development and function of the GABAergic parvalbumin-positive interneurons, involvement in acetylocholine neurotransmission, or neuroprotection in the nigrostriatal pathway [164]. The abovementioned functions are due to various properties exerted by selenium, including antioxidant effects, involvement in calcium homeostasis, and brain cholesterol metabolism. It was shown that impaired levels of selenium in the diet (namely, too low supply) might lead to greater anxiety, depressive symptoms, and tiredness [165]. There are discrepancies regarding the association between serum selenium levels and gender; some studies indicate that there is no relationship, while others claim that it is either males or females who present greater selenium levels in depression [166]. However, there is a clear relationship between selenium intake and age since it was noted that selenium deficiency increases in proportion to age, leading to the conclusion that selenium supplementation should also be increased with age [167].

Given its significance in the CNS, there is a hypothesis that maintaining adequate selenium status may be linked to a reduced risk of depression and depressive symptoms. Preclinical studies on animals have suggested that selenium may act as an antidepressant by influencing the dopaminergic and serotonergic systems [168].

The observed low serum selenium concentration in patients with MDD compared to healthy individuals, as reported by Islam et al., further supports the notion that deficient selenium concentrations could be a risk factor for depressive mood, anxiety, and cognitive function decline, with implications on antioxidant pathways and reduced enzyme activity [169]. Further, it was observed that pregnant and postpartum women with low selenium intake are at a greater risk of experiencing postnatal depression; high selenium intake during pregnancy might significantly reduce the risk of experiencing depressive symptoms in pregnant women [170].

Furthermore, selenium’s modulation of thyroid hormones and neurotransmitters, such as dopamine and serotonin, also seems to be related to depression [171,172]. Dysregulation of thyroid function due to selenium deficiency may increase the risk of mood disorders [173]. Additionally, selenium’s association with brain-derived neurotrophic factor levels influences neuroplasticity and may play a role in the pathophysiology of depression [174].

The influence of selenium on depression appears to be affected by dietary intake and biomarkers at adequate levels [118]. Studies analyzing selenium consumption in different ways have yielded varied results, reinforcing the hypothesis that the connection between selenium and depression is dependent on dietary intake and/or biomarkers at adequate levels [118]. Observational and interventional studies have reported inconsistent findings regarding the role of selenium in depression, with some showing a significant negative relationship between selenium levels and depression risk, while others did not find such a relationship [175,176,177,178,179]. Additionally, selenium supplementation has shown mixed effects on depression symptoms in clinical trials [180,181,182]. Despite the presence of various outcomes, selenium supplementation is believed to significantly reduce the symptoms of MDD, making it a possible adjuvant therapy for patients with MDD. So far, selenium dosages used to investigate the relationship between selenium supplementation and MDD symptoms have varied between 100 and 200 μg [166]. Selenium supplementation has a significant impact on the alleviation of depressive symptoms via its anti-inflammatory properties, facilitating the functioning of various antioxidant enzymes such as thioredoxin reductases or GPx, lowering the intensity of oxidative stress, or modulating neurotransmission. Even though further research in this matter should focus on establishing supplementation strategies and dietary recommendations for patients with MDD.

To better understand the relationship between selenium and depression, the use of appropriate methodologies to measure selenium concentrations in serum is crucial. Techniques such as atomic absorption spectrometry, molecular, atomic fluorescence spectrometry, and inductively coupled plasma-mass spectrometry have been employed [183,184,185,186]. However, differences in sample preparation and detection capabilities among these methods may contribute to the heterogeneity observed in the study findings [187,188].

Furthermore, factors like sex and age may influence serum selenium concentrations, with conflicting reports on the association between gender and selenium levels [189,190,191,192]. Age-related changes in selenium distribution, hormonal status, and dietary habits may also play a role in affecting selenium concentration through different stages of life [193,194,195]. Additionally, maternal selenium levels during pregnancy and lactation can impact the risk of postpartum depression, further highlighting the importance of maintaining proper selenium intake during these periods [196].

Selenium’s role in the brain’s antioxidant system, thyroid hormone metabolism, and neurotransmitter regulation suggests its potential influence on mood disorders such as depression. However, the relationship between selenium and depression is complex and influenced by various factors, including dietary intake, biomarkers, sex, and age. Further research is needed to elucidate the precise mechanisms underlying this association and optimize selenium’s potential therapeutic applications for depression.

## 7. Iron

Iron is the most abundant metal in a human brain, being responsible for the synthesis of various neurotransmitters (including noradrenalin, dopamine, adrenaline, and 5-hydroxytryptamine), neuronal myelination, or proper functioning of the mitochondria. Low iron levels might be associated with depressive symptoms because of the modulation of the abovementioned neurotransmitters. There are several neurological and psychiatric conditions associated with iron deficiency; except for MDD, which is the major scope of this article, we can distinguish ADHD, Alzheimer’s disease, Parkinson’s disease, anxiety, or schizophrenia [165,197]. Iron deficiency has been associated with depression-like symptoms such as fatigue, agitation, apathy, and poor concentration [198]. An association was found between iron deficiency anemia and a history of depression and stress symptoms [198]. Postpartum depression risk has also been linked to iron deficiency anemia or depletion of iron stores in mothers after giving birth [199]. For instance, Albacar et al. reported a strong association between ferritin levels 48 h after childbirth and postpartum depression [200]. Based on these findings, assessing serum iron and ferritin levels for potential iron deficiency and administering iron supplements have been proposed as effective interventions to improve depressive symptoms [200]. However, caution must be exercised to avoid iron overdosing, as excessive iron administration may lead to oxidative stress, increased blood viscosity, and an impaired systemic response to inflammation and infection, potentially leading to various diseases [201].

Studies have reported a higher prevalence of anemia in patients with psychiatric disorders, including depression, compared to the general population [202]. Nevertheless, some authors have not found a significant association between blood hemoglobin levels and depression [203].

Limited studies on iron-deficiency anemia (IDA), the most common nutritional deficiency, have shown an association between IDA and depression severity, particularly with somatic symptoms [204]. Sleep quality, anxiety, and depression scores were found to be worsened in patients with IDA compared to healthy controls [205]. Additionally, depressive symptoms were associated with serum iron deficiency status in elderly individuals [206].

The relationship between poor nutrition and depression has been suggested, especially in women of childbearing age who are susceptible to depletion of nutritional factors, including iron [207,208]. Hemorrhage during the postpartum period has been linked to anemia, iron deficiency, and depression [209]. Low blood hemoglobin levels, iron status variables, and plasma ferritin levels have been associated with postpartum depression [210]. However, a sex difference has been observed, as the association between low serum ferritin levels and increased depression prevalence was significant in men but not women in a middle-aged population [211].

Micronutrients, including iron, have been reported to individually affect the brain and mood [212,213]. A recent meta-analysis indicated a negative association between dietary iron intake and the risk of depression [214]. Iron plays a crucial role as a cofactor for enzymes responsible for monoamine synthesis (i.e., dopamine and serotonin) in depression [215]. In cases of comorbid IDA and depression, addressing iron deficiency prior to the use of antidepressants targeting the monoamine system may prove beneficial [215]. The relationship between iron status and brain functions has been suggested, providing further insight into the potential role of iron in depressive disorders [40,216].

Iron deficiency and anemia have been associated with depressive symptoms and postpartum depression risk. Iron supplementation may be an effective strategy to improve depression in individuals with iron deficiency, although careful dosing is essential to avoid potential adverse effects. Understanding the intricate relationship between iron status and depression is crucial for developing targeted interventions for mood disorders. Identifying individuals with depression who present iron deficiency can lead to more targeted interventions, such as adding proper iron supplementation to patients with MDD. What’s clinically important in this matter is careful dosing and monitoring of iron levels to avoid potential adverse effects such as hypothyroidism, pancreatic islet cell damage, liver cirrhosis, or even liver damage. The role of proper diet and lifestyle factors should be considered to maintain adequate iron levels; this matter is primarily important with regards to those individuals who are susceptible to nutritional deficiencies since imbalanced iron levels might facilitate depressive symptoms.

## 8. Copper

Copper, as a trace metal, holds significant importance in various physiological processes, including erythropoiesis, cellular respiration, peptide amidation, and hormone biosynthesis, making it vital for essential body functions [217]. There is an increasing number of studies indicating that copper might be involved in neurotransmission and facilitate information processing in the human brain [218]. In the context of depression, copper’s involvement in the monoaminergic approach is noteworthy, particularly its role in the conversion of dopamine to norepinephrine [219]. This conversion relies on copper ions interacting with dopamine β-hydroxylase [220]. Copper is crucial for the proper functioning of the dopamine beta-hydroxylase, which is involved in the conversion of dopamine to norepinephrine in noradrenergic and adrenergic neurons or adrenal chromaffin cells [221]. The relationship between oxidative stress and inflammation has also been established in depression [222]. Copper concentration is closely linked to oxidative stress processes and the properties of antioxidant enzymes, which may contribute to the development of depression [223,224]. Copper stimulates the production of ROS that are involved in oxidative stress that damages DNA, proteins, and lipids [225]. Further, it should be noted that ceruloplasmin, a major copper transporter in the human organism, was observed to be increased in patients suffering from mood disorders [226]. Although the role of ceruloplasmin in depression remains uncertain, elevated serum levels of ceruloplasmin may indicate the potential significance of copper ions in mood disorders [227,228,229]. It was shown that high copper levels induce NMDA receptor dysfunction, and its altered functioning leads to the onset of cognitive deficits in MDD [117]. Elevated copper concentrations also lead to dysfunctions of the AMPA receptor function, inducing disturbances of glutamatergic transmission, which constitute the basis of the Glu hypothesis of depression [230,231,232]. The abovementioned processes might significantly contribute to the development and progression of MDD. Apart from MDD, imbalances in the copper levels might be a reason for the occurrence of such neuropathic diseases as Alzheimer’s disease, Wilson’s disease, or Menkes disease [233].

Furthermore, researchers are investigating the interaction between copper and the NMDA/Glu pathway, particularly in relation to ketamine, which acts as an NMDA receptor antagonist [231,232,234,235,236,237,238]. The evidence suggests a potential synergistic interaction between copper and ketamine, potentially holding therapeutic relevance for mood disorders. Nevertheless, further research on the relationship between copper and ketamine in depression treatment is warranted.

While the role of copper in depression is complex and not fully elucidated, research indicates that copper plays a crucial role in the proper development and functioning of the CNS [54]. Some studies have observed no significant differences in serum copper concentrations between patients with depression and healthy controls [230]. Conversely, other research has shown higher copper levels in depressed patients [239]. The discordant results may be attributed to factors such as differences in patient characteristics, sample size, or measurement methods.

Copper’s involvement in depression is multifaceted, encompassing interactions with neurotransmitters, modulation of receptors, and effects on oxidative stress and inflammation pathways. Although the precise role of copper in depression remains incompletely understood, its potential significance in mood disorders warrants further investigation. The conflicting findings in existing research highlight the need for more comprehensive and standardized studies to gain a better understanding of the relationship between copper and depression.

## 9. Aluminium

Although aluminum is present in various bodily tissues, its physiological role remains unknown. Even though what should be considered is the fact that aluminium presence in human organisms is rather associated with adverse effects since it has been shown to be toxic for humans. It was demonstrated that the brain is very prone to aluminium toxicity, leading to neurodevelopmental and neurodegenerative disorders [240]. Neurotoxicity can occur as a result of the accumulation of aluminum in the body, which can happen by ingestion or inhalation [241]. Excessive aluminium exposure might be prevented by various solutions, including the reduction of aluminium foil usage, filtration of drinking water, using non-aluminium cosmetics, avoiding canned food, and choosing high-quality supplements. Regarding neurotoxicity associated with aluminium exposure, it appears mainly via aluminium-facilitated mechanisms such as oxidative stress, mitochondrial dysfunction, apoptosis, neuroinflammation, and microglial activation, as well as reduced synaptic plasticity and neurotrophin production. Further aluminium exposure is associated with impaired neurotransmitter metabolism and signal transduction affecting glutamatergic-GABAergic, cholinergic, dopaminergic, and serotoninergic systems [240]. Aluminium toxicity might be associated with such disorders as Alzheimer’s disease, alcohol use disorder, ASD, Parkinson’s disease, dialysis encephalopathy, or multiple sclerosis [242]. There have been reports indicating that those employed in the aluminum industry are experiencing mental health issues, including cognitive decline, memory impairment, depression, and anxiety [243]. In the experimental group subjected to aluminum exposure, 7% of individuals exhibited symptoms of severe depression, 11% displayed symptoms of moderate depression, and 25% showed signs of mild depression. In contrast, the control group showed a prevalence of mild depression in 20% of participants. Therefore, the findings of this study indicate a statistically significant disparity in depression levels between the participants who were exposed to aluminum and those who served as controls. The findings of a separate investigation indicated a statistically significant elevation (*p* < 0.001) in the concentrations of aluminum among individuals diagnosed with MDD in comparison to the control group [244]. Research shows that elevated levels of aluminum can hinder the functioning of dopamine beta-hydroxylase, resulting in reduced synthesis of noradrenaline. There is evidence suggesting that a shortage of noradrenaline is associated with the onset of depression [245]. Hence, the conclusions drawn from the aforementioned studies indicate a potential correlation between elevated levels of Al and an augmented susceptibility to MDD. Further investigation is necessary to elucidate the involvement of aluminum (Al) in the pathophysiology of mental diseases, as the existing evidence is significantly constrained.

There is evidence suggesting a correlation between oxidative stress and a wide range of mental diseases, including MDD. This association is attributed to the vulnerability of the human brain, which is regarded as extremely susceptible to oxidative stress [246,247]. Therefore, it is assumed that the mitigation of neurotoxicity caused by aluminum can be achieved through the utilization of potent antioxidants such as lithium [248], bacopa monniera, L-deprenyl [249], or trihydroxy piperlongumine [250].

## 10. Cadmium

Cadmium is known to have a significant impact on the development and progression of depression [251]. The primary source of cadmium intake in the non-smoking population is food consumption. This is because some crops, such as potatoes, cereals, and vegetables, can absorb cadmium from the soil [252]. Conversely, the smoking population is exposed to greater concentrations of cadmium through the consumption of tobacco products [253]. The harmful effects of cadmium are mostly attributed to its presence as free cadmium ions, which can connect with atoms of sulphur, hydrogen, and oxygen. This interaction disrupts numerous metabolic cycles within the organism [254]. For example, the binding of cadmium ions to mitochondria has the potential to impede the processes of respiration and oxidative phosphorylation [255]. Cadmium has been found to elicit neurotoxic effects by many mechanisms, such as its disruption of the blood–brain barrier [256], augmentation of oxidative stress, interference with zinc and calcium-dependent cellular activities, stimulation of metallothionein, and initiation of apoptosis [257,258]. It was shown that cadmium passes through the blood–brain barrier and tends to accumulate in the CNS, inducing neurotoxicity that is presented by a vast number of symptoms such as attention deficits, memory impairments, learning disabilities, headaches, vertigo, olfactory dysfunctions, or peripheral neuropathy [256]. Further, it was shown that excessive cadmium levels might lead to imbalances of excitation-inhibition in synaptic neurotransmission [259]. Research has demonstrated that the insufficiency of essential elements, such as zinc and iron, can lead to an elevation in the absorption and accumulation of cadmium, contingent upon factors such as age and sex [260,261,262]. Additionally, there is evidence linking cadmium exposure to synaptic dysfunction, specifically by inhibiting the absorption of Glu dependent on sodium ions and blocking the activity of voltage-gated calcium channels [263]. Cadmium has the potential to contribute to the development of depression by negatively affecting the monoaminergic neurotransmission system [262,264,265]. Cadmium is capable of negatively impacting the CNS by initiating vascular damage through endothelial oxidative stress [266]. It was suggested that cadmium might be associated with the pathophysiology of such psychiatric conditions as bipolar disorder or schizophrenia, primarily via the disruption of mitochondrial functions due to cadmium toxicity [251]. Further, various studies have indicated that this oxidative stress is considered a risk factor for depression. It is suggested that alterations in dopamine, norepinephrine, serotonin, or thyroid hormone levels induced by cadmium exposure may be associated with the development of depression [267,268]. The tentative acceptable weekly intake of cadmium was determined by the Scientific Committee on Food to be 7 μg/kg body weight [269]. A study by Berk et al. found a positive correlation between higher levels of cadmium and depression, as indicated by the adjusted prevalence ratio (PR) of 1.48 (95% CI 1.16 to 1.90) [270]. Scinicariello and Buser found that those in the highest quartile of cadmium had significantly increased chances of experiencing depressive symptoms compared to those in the lowest quartile of cadmium [264]. It is noteworthy that the concentration of cadmium in the bloodstream of individuals who smoke tobacco can be up to four or five times greater in comparison to those who do not engage in smoking [271,272,273]. This observation can be attributed to a substantial association between the quantity of cigarettes consumed, the duration of tobacco smoke exposure, and the Cd level in the body [274].

Concerning the subject of cadmium, it is noteworthy to acknowledge the emergence of ketamine as a novel pharmacological intervention for the treatment of depression. Ketamine functions as an antagonist of the NMDA receptor, hence augmenting the release of presynaptic Glu [275]. Another therapeutic approach involves the utilization of vitamin B3, which possesses notable antioxidant characteristics [276]. Consequently, it may serve as a potential safeguard against oxidative stress elicited by the presence of heavy metals. Multiple studies concluded that the consumption of vitamin B3 through one’s diet is correlated with a decreased likelihood of experiencing depression [277,278]. In another study, it was observed that individuals diagnosed with depression exhibited significantly reduced levels of vitamin B3 in comparison to the control group [279].

There was an observed correlation between elevated levels of serum cadmium and an elevated likelihood of experiencing depression. Notably, it was shown that the risk of depression decreased significantly when there was an increase in the consumption of vitamin B1, B3, or total vitamin A. The disruptive effects of cadmium can also be addressed by the use of ketamine, which has recently emerged as a novel therapeutic intervention for depression.

## 11. Lead

The global prevalence of dangers associated with human exposure to heavy metals has escalated simultaneously with the processes of urbanization and industrialization [280]. Lead exposure can arise from multiple sources, such as industrial pollution affecting the air, water, and soil [281], the presence of lead paint, imported pottery, dust, batteries, and occupational sources of lead [282]. The substance in question is a heavy metal known for its significant toxicity, which has detrimental effects on several enzymes and structural proteins within the human body when it accumulates. Lead has been found to have an impact on many enzymes involved in the respiratory chain, glycolysis route, and hem synthesis. Consequently, this leads to disruptions in the metabolic transformations occurring within cells, including the regulation of energy activities as well as the synthesis of proteins and nucleic acids [283]. The presence of lead has been found to affect various pathways that are recognized to play a role in the development of depression, including neurogenesis and apoptosis, oxidative stress and glutathione, Glu, calcium, and calmodulin, as well as neurotransmitters like acetylcholine [284,285,286,287]. It was shown that increased lead levels might be associated with MDD, or panic disorder [288]. A study demonstrated that those with blood lead levels in the highest quintile (more than 2.1 μg/dL) exhibit 2.3 times the likelihood of severe depressive disorder (95% CI 1.13–4.75) compared to those in the lowest quintile [288].

A meta-analysis [274] on the subject matter revealed that at least nine studies have shown evidence of a correlation between depressive symptoms and increased levels of blood lead (bPb) [288,289,290,291,292,293,294,295,296]. Numerous authors have documented that exposure to Pb has been observed to interfere with catecholaminergic systems, resulting in the manifestation of depression and anxiety disorders [297,298,299]. Research conducted on animals has demonstrated that prolonged exposure to lead can result in a reduction of serotoninergic activity in several parts of the brain, such as the nucleus accumbens, frontal cortex, and brainstem [300]. In a study conducted by Rajan et al., it was observed that the hypothalamus had the greatest concentrations of Pb [301].

There are multiple mechanisms via which vitamin B1 interacts with Pb to prevent tissue buildup, thereby preventing the manifestation of clinical symptoms and mortality. The authors have put forth the proposition that therapeutic dosages of vitamin B1 could potentially serve as a beneficial measure in the prevention and treatment of individuals or animals who have been exposed to elevated surrounding levels, thereby mitigating the adverse consequences associated with its toxicity. According to the findings of Reddy et al. [302], it was postulated that Pb has the potential to interact with the pyrimidine ring of vitamin B1, resulting in the solubilization of the vitamin at pH levels commonly found in the human body. Additionally, the researchers revealed that therapy with vitamin B1 can decrease Pb concentrations in many bodily tissues, including the kidneys, blood, and bones.

## 12. Mercury

Mercury, being a toxic heavy metal, has the potential to accumulate within the human body as time progresses. Various factors can contribute to the exposure of individuals to mercury, including but not limited to cosmetic preparations, fossil fuels, air, polluted waste, and food, notably shellfish and fish that have been contaminated [303,304,305]. Mercury has been widely studied in many research studies since it was observed that it tends to accumulate within the CNS in high amounts, leading to neurotoxicity [306]. The ingestion of mercury is commonly considered an indicator of seafood consumption, which might potentially be the influential factor, or it might serve as an indicator for other variables such as socioeconomic level and intake of omega-3 fatty acids [307,308]. Consequently, the possible adverse impact of mercury on depression prevalence may be counterbalanced by the preventative advantage associated with the consumption of fish, which has been correlated with a reduced incidence of depression [309]. This finding explains the observed correlation between higher levels of mercury and the negative association with depression [270]. Additionally, a study conducted by Kim et al. [310] revealed a positive correlation between elevated levels of serum mercury and the incidence of depression among Korean women, particularly those with lower seafood consumption. Although three investigations could not establish a correlation between elevated levels of total serum mercury and an elevated likelihood of experiencing depression among adults in the United States [311] and in Korean adults [312], as concluded by Nguyen et al. [277]. The negative effects of mercury exposure arise from its ability to traverse the blood–brain barrier, leading to neurotoxic symptoms [313]. In particular, the presence of inorganic mercury, notably methylmercury, can induce neuropsychiatric symptoms by instigating oxidative stress within the CNS [314]. Based on the results of the previously described research, consumption of fish obtained from uncontaminated sources has been shown to provide a protective effect against exposure to Hg. An additional approach to mitigating the symptoms of depression associated with mercury exposure is to enhance the intake of vitamin B1. Research conducted on the Korean population has demonstrated an inverse correlation between vitamin B1 consumption and depression [277]. A separate study revealed that the administration of vitamin B1 over several weeks resulted in a notable amelioration of depressive symptoms among a cohort of 80 older women [315]. This finding instills optimism in individuals afflicted with geriatric depression as well as in the rest of the population.

## 13. Arsenic

Arsenic is a well-established toxic substance with a long-standing history of usage and diverse applications [316]. The biological purpose of arsenic is not yet understood, and instead, it is associated with detrimental health impacts [317,318]. Arsenic exposure might be via various routes, including drinking water, industrialized and wood preservatives, diet, air, smoking, or cosmetics. Neurotoxicity associated with arsenic exposure is associated with numerous mechanisms, namely increased levels of ROS, cytotoxicity, cellular DNA damage, and chromosomal aberrations. Further, arsenic alters the levels of dopamine, serotonin, Glu, GABA, and norepinephrine [319]. The mental health consequences resulting from arsenic poisoning are significant [320]. It was demonstrated that arsenic toxicity might be associated with such psychiatric conditions as MDD or anxiety [321]. The neurological impacts associated with arsenic exposure encompass a range of symptoms, including but not limited to depression, sleeplessness, anxiety, and cognitive deficits [322]. The Asian region exhibits a higher susceptibility to arsenic contamination in comparison to other global regions, thereby rendering it the primary focus of extensive research efforts [320]. A research paper by Mukherjee et al. [323] examines the impact of chronic low-level arsenic exposure on Indian women. The findings reveal a significantly higher prevalence of depressive symptoms among individuals exposed to higher levels of arsenic in groundwater (28.3 ± 13.51 mg/L) compared to the control group exposed to lower levels (2.72 ± 1.18 mg/L). The prevalence rates were 39.8% and 19.9%, respectively. It is noteworthy to remark that the recommended threshold for the concentration of arsenic in typical drinking water is 10 μg/L [324]. Furthermore, a cross-sectional investigation conducted in two villages located in Inner Mongolia, China, revealed that individuals residing in an area impacted by arsenic contamination have a higher propensity for experiencing distress symptoms in comparison to those residing in an area free from arsenic contamination [325]. This association persists even after controlling for factors such as age and sex. According to a study conducted in the United States [326], there was a notable association between individuals residing in areas with mid-range arsenic concentrations in their wells (ranging from 2 μg/L to 10 μg/L) and a higher likelihood of reporting depression compared to respondents residing in areas with the lowest arsenic concentrations (below 2 μg/L). This finding provides additional evidence regarding the significant impact of arsenic on mental well-being. The presence of arsenic, in conjunction with cadmium, lead, and mercury, can also have detrimental effects on individuals’ health, potentially contributing to the onset of depression. The researchers conducted an observation and found that the presence of arsenic had an impact on 19 genes that are associated with the development of depression. The genes were identified as being situated on chromosome 1q31.1 (chr1q31.1) [327]. The genetic expression of SOD1, IL6, PTGS2, PON1, BDNF, and ALB was modified by the presence of a mixture of heavy metals. This alteration underscores the significance of oxidative stress, pro-inflammatory cytokines, paraoxonase activity, neurotrophic factors, and antioxidants in relation to depression. Moreover, it suggests the potential for therapeutic interventions targeting these specific genes in the treatment of depression [327]. The presence of arsenic leads to a substantial elevation in oxidative stress levels, resulting in detrimental consequences. However, the administration of powerful antioxidants, such as selenium, has the potential to impede these deleterious effects [328]. Histopathological investigations have demonstrated the reversal of hippocampal degeneration in rats treated with arsenic with the administration of selenium. The findings from this study suggest that the concurrent administration of selenium at doses of 0.175 and 0.35 mg/mL/kg may have a protective impact against arsenic-induced toxicity while also exhibiting potential to counteract behavioral abnormalities by restoring the activities of SOD, CAT, and GPx while also lowering oxidative stress induced by arsenic [328]. Furthermore, previous studies demonstrated that the harmful effects caused by arsenic exposure during the perinatal period can be reversed with the administration of persistent fluoxetine medication. This treatment has been found to restore the individual’s ability to cope with depression by engaging in a neurogenic process [329].

## 14. Calcium

Calcium is a prevalent cation that is found in abundance within the human body. Its presence is essential for the management of neurotransmitter homeostasis as well as for the facilitation of neuromuscular excitability [330]. Additionally, calcium serves as a cofactor for various enzymes, including those involved in the blood coagulation cascade [224]. Calcium plays a significant role in the control of various processes associated with affective disorders. These activities include the synthesis, release, and receptor responsiveness to neurotransmitters, as well as the maintenance and termination of the action potential. Furthermore, calcium is involved in the neuronal memory of prior stimulation [331]. Dysregulation of calcium levels and associated signaling pathways might be associated with the development of such psychiatric disorders as MDD, schizophrenia, or bipolar disorder [332].

Multiple studies indicated a lack of correlation between blood calcium levels and depression [330,333,334]. However, some research [335] reveals that individuals with chronic depression exhibit elevated mean diurnal serum Ca and Mg levels. Furthermore, the severity of depression in chronic patients is found to be positively associated with serum Ca and Mg levels. Specifically, the serum calcium level in individuals with acute depression was measured to be 2.41 ± 0.09 mmol/L, whereas control subjects exhibited a level of 2.25 ± 0.05 mmol/L. The researchers also reported a correlation between slowness of movement and the peak of diurnal serum calcium in the acute condition, which is regarded as a consistent observation. According to a study conducted by Islam et al. [169], individuals diagnosed with MDD had a statistically significant reduction in blood calcium levels of 94.91 ± 0.85 mg/L compared to control subjects—105.06 ± 1.05 mg/L (*p* < 0.05). Based on the findings of Galeotti et al.’s study [336], which demonstrated that the administration of TMB-8, a substance that inhibits the release of intracellular calcium, resulted in a decrease in immobility time during the forced swim test, performed with rats, and yielded a response comparable to that of tricyclic antidepressants such as amitriptyline and clomipramine, Młyniec proposed a hypothesis [224]. The hypothesis proposes that such collective data strongly indicate the presence of disrupted intracellular calcium dynamics in certain affective disorders. Furthermore, it suggests that the regulation of calcium levels through the use of calcium channel blockers may be an effective approach to reducing depressive-like symptoms, as supported by both animal and human studies. It implies that the pharmaceutical inhibition of calcium entry into cells may have a potential therapeutic effect on depressive-like symptoms. Additionally, it is proposed that a dietary approach with a higher intake of magnesium relative to calcium, at a ratio of 1:2, could possibly yield similar benefits. The primary barrier to viewers’ effectiveness in managing depression seemed to be persistent and excessive consumption of dietary calcium and scarce intake of magnesium, although the consumption of Mg has been found to reduce symptoms of depression [160]. Moreover, an excessive intake of calcium hinders the absorption of magnesium in the gastrointestinal tract. The potential depletion of magnesium ions may indeed impair the regulation of calcium ions within neuronal channels, leading to the occurrence of pathogenic calcium ion cascades [160]. Paul proposed that the implementation of strategies aimed at decreasing pathological neuronal calcium ion influx, to subsequently reduce the resultant pathological nitric oxide neuronal production, could potentially yield antidepressant effects [337]. Statistical data indicates that approximately 7.6% of individuals diagnosed with psychiatric disorders experience challenges related to their dietary status [338]. A study noticed depressive symptoms in elderly Japanese women who had calcium shortages, comparing them to healthy controls who consumed an average of 350.93 ± 124.13 mg/1000 kcal of calcium. The participants with depressive symptoms had an average calcium intake of 308.59 ± 113.88 mg/1000 kcal. In contrast, no significant disparity in mineral intake was seen between male participants exhibiting depressive symptoms and those who did not exhibit such symptoms. The findings of this study indicate that there is a significant association between gender and the impact of mineral intake on depression symptoms; it was suggested that calcium deficiencies and their association with the onset and progression of depressive symptoms are more visible in females [339]. This suggests that individuals diagnosed with depression were shown to have a dietary pattern characterized by low nutritional quality, which has been associated with the development and exacerbation of depressive symptoms [340].

Based on those findings, regulating the diet of a depression-affected person should not be overlooked and might even be considered the first line regarding patients with MDD.

## 15. Manganese

Manganese is an essential trace element in the human diet due to its indispensable role in regulating various physiological processes, including immunological function, blood sugar control, cellular energy metabolism, reproductive function, digestion, wound healing, and defense against ROS [341]. The absorption of manganese can occur via the gastrointestinal system or by inhalation [224]. The potential for exposure to Mn particulate matter arises from inhalation in the vicinity of mining operations and establishments that utilize manganese. Occupational hazards pose a greater risk to welders, miners, and processors. Manganese passes through the blood–brain and blood–cerebrospinal fluid barriers, which are the major routes for manganese occurrence in the CNS [342]. Mechanisms of manganese toxicity are not fully understood yet; however, some of the neurotoxicity occurring in the CNS might be because of the involvement of manganese in the alterations of dopamine, GABA, Glu or acetylocholine levels or disturbances induced in mitochondrial functioning [343].

According to clinical neuropsychologists, the mental health of the bridge welders was found to be favorable prior to engaging in welding activities. A significant proportion of the participants, specifically 63% and 81%, respectively, indicated the presence of clinical manifestations associated with depression and anxiety after engaging in job and welding activities without adequate protective measures [344]. The presence of manganese has been observed to serve as a catalyst in the Fenton reaction, leading to the development of neurotoxicity, oxidative stress, and neurodegenerative mechanisms [345,346]. The Fenton reaction is the reaction that leads to the formation of free radicals in acidic conditions in the presence of iron ions. Elements involved in the Fenton reaction, such as hydroxyl radicals, hydrogen peroxide, and other radical species, are highly toxic due to their ability to disrupt DNA functioning; they are also involved in the oxidation of proteins and amino acids, as well as the lipid peroxidation of membrane fatty acids. Subsequent research revealed a positive correlation between elevated cumulative manganese exposure and an increased propensity to experience psychological distress associated with depression [347]. According to the recommendations provided by the Agency for Toxic Substances and Disease Registry, the acceptable range for manganese blood levels is between 4 and 15 g/L [348]. Manganese predominantly resides in hepatic tissue, yet it possesses the ability to cross the blood–brain barrier and amass within several regions, including the basal ganglia, globus pallidus, hypothalamus, nucleus caudatus, corpus pineale, and putamen [349]. The early diagnosis of manganese neurotoxicity can be facilitated by the utilization of the pallidal index [350]. Remarkably, a cross-sectional study conducted on Japanese employees revealed a significant association between insufficient intake of zinc, copper, and manganese and the presence of symptoms related to anxiety and depression, independent of other dietary, lifestyle, and occupational factors [351]. A research investigation conducted on Spanish schoolchildren revealed that the average intake of Mn was comparatively lower among pupils exhibiting depressive symptoms (2.63 ± 1.26 mg per day) as opposed to those who did not manifest such signs (3.09 ± 2.38 mg per day) [351]. Another study found that there was a negative correlation between manganese consumption and depression symptoms among Japanese women during pregnancy [352]. Depressive symptoms during pregnancy were found in 19.3% of the 1745 study participants. Manganese’s median daily energy-adjusted consumption was 3.6 mg. MnSOD activity may be reduced owing to manganese deficiency, which may contribute to the development of depressive symptoms [353]. As a result, increased manganese consumption may be expected to reduce the prevalence of depressive symptoms through boosting MnSOD activity. In contrast, a Mexican cohort study has established a correlation between elevated blood manganese levels during the 3rd trimester of pregnancy and the occurrence of postpartum depression [354]. It is worth noting that the main source of manganese exposure may not originate from the environment but rather from internal sources through the remobilization of Mn tissue stores within the body. The observed correlation between exposure to manganese in the environment and the manifestation of depressive symptoms in professional environments aligns with existing scientific research on the relationship between manganese and depressive symptoms. Through the integration of research conducted on Japanese pregnant women, welders, and women in Mexico, it can be deduced that, just as it has been observed by Młyniec, there exists a potential correlation between manganese levels and depression, which can manifest either in an inverse or positive manner [224]. MnSOD is a prominent enzymatic antioxidant that plays a crucial role in safeguarding cellular integrity against oxidative stress within the mitochondria. The process involves the catalysis of superoxide to generate hydrogen peroxide and oxygen. Individuals diagnosed with bipolar disorder in the depressive phase exhibited reduced levels of SOD-2 compared to a control group consisting of individuals without mental health conditions. After 30 days, the administration of fluoxetine increased the activity of SOD-2 [355]. Existing research indicates a correlation between the lowered concentration of SOD-2 and the decreased volume of the prefrontal cortex and hippocampus in individuals with severe depression [356].

There has been a comparatively limited amount of research conducted on the examination of manganese levels in the brains of individuals diagnosed with MDD. Further investigation is warranted to establish the threshold of manganese exposure in relation to the heightened susceptibility to depression associated with manganism. Additional research is required to investigate the relationship between manganese deficiencies and MDD. Exploring the levels of Mn that result in benefits for the population should be at the forefront of new studies.

## 16. Chromium, Nickel, and Phosphorus

The biological function of nickel in the human body is still unclear. Nickel exposure might either stimulate or inhibit the release of dopamine, as well as inhibit Glu NMDA receptors. Animal models have shown that nickel could be associated with alterations in motor activity, memory, and learning impairments, along with the presence of anxiety and depressive-like symptoms [357]. Nickel stimulates neurotoxicity primarily via the induction of oxidative stress, which leads to impairments of neuronal functions and altered neurotransmission. The abovementioned processes, primarily the production ROS and oxidative stress stimulation, are associated with the onset and progression of various neurodegenerative and neuropsychiatric conditions such as Alzheimer’s and Parkinson’s diseases, MDD, anxiety, or schizophrenia. It is also hypothesized that chromium might influence dopamine levels indirectly via serotonin release [358]. So far, there is no information on the effects of nickel on mental disorders. It was found that nickel values were statistically lower in MDD patients compared to healthy controls. Therefore, these results may be a particularly important and reliable source for understanding the possible role of nickel in psychiatric disorders, especially depression. Further studies are needed to elucidate the role of nickel in the pathogenesis of psychiatric disorders [244].

It has been found that serum chromium levels were significantly higher in MDD patients than in healthy individuals. These results strongly suggest an association between Cr exposure and an increased risk of MDD. Chromium, as a toxic substance, can trigger oxidative stress in the human body and eventually lead to cellular disorders. Therefore, it can be hypothesized that the high chromium values estimated in this work are related to the pathogenesis and etiology of mental disorders [244].

Furthermore, this study revealed some significant disturbances in serum levels in Ni-Cr-MDD patients, which can be used to monitor disease progression. The lack of chromium and the increase of nickel may indicate their partial participation in the perturbation process [244].

There was no difference in serum phosphorus (P) levels between depressed patients and healthy subjects [359].

Currently, there is no information about other elements that could possibly be associated with MDD patients.

## 17. Conclusions

The results of the current research indicate that there are various relationships between the status of the metal content of the body and the outcome of patients with MDD. It was shown that a combination of antidepressant therapy and supplementation of such elements as zinc, magnesium, selenium, or iron significantly improves the clinical outcome of patients with MDD. These findings suggest that supplementation with the aforementioned elements could enhance the effects of antidepressant drugs, and the control of their blood concentrations could potentially act as biological markers for the assessment of MDD severity. Except for the elements for which deficiencies might lead to the progression of depressive symptoms, it was shown that excessive cumulation of such elements as aluminium, cadmium, or mercury could also worsen the course of the disease. Such discrepancies in the studies evaluated in this review might be because of individual patients’ characteristics or different measurement methods. Accurate analysis of trace elements in various samples is a challenging task that requires highly sensitive techniques such as AAS or ICP-MS. What is most challenging and might lead to different results include matrix effects, contamination, and instrument drift. Knowledge about the alterations in the content of chosen elements in the abovementioned samples, along with the patient’s serum that was already investigated in this paper, could lead to improvements in the therapeutic approach towards patients with MDD, leading to more promising patient outcomes. The content of metals in chosen samples might potentially act as either prognostic or predictive biomarkers of MDD; however, more research in this area is needed.

## Figures and Tables

**Table 1:** Physiological ranges and biological functions of trace elements in the human organism.

Element	Healthy Ranges: Female	Healthy Ranges: Male	Biological Functions	References
Selenium (Se)	81.06–164.75 (121.05) μg/L		Selenium (Se) is an essential trace element that plays a crucial role in various biological processes. Maintaining adequate selenium levels is important for overall health and well-being. Low selenium status has been associated with several health issues, including an increased risk of mortality, compromised immune function, and cognitive decline. Selenium is a key component of selenoproteins, which are essential for various physiological functions, including antioxidant defense, immune response, thyroid hormone metabolism, and DNA synthesis. Adequate selenium levels are important for supporting the body’s immune system, protecting cells from oxidative damage, and promoting healthy cognitive function. However, selenium is a “dual-surface” element, meaning that both deficiency and excess can be harmful. Excessive selenium intake can lead to a condition called selenosis, which is characterized by symptoms such as gastrointestinal disturbances, hair and nail brittleness, skin rashes, and even neurological issues.	[37]
Iron (Fe)	11–29 µmol/L (serum)	14–32 µmol/L (serum)	Iron (Fe) is an essential trace element for all living organisms due to its critical role in various physiological processes. It is a crucial component of hemoglobin, the protein responsible for storing and delivering oxygen in red blood cells. Fe is also required for the synthesis of myoglobin (a protein found in muscles), catalase, peroxidase, and cytochromes, which play important roles in various cellular functions. Fe is a vital component of numerous proteins involved in DNA synthesis and cell proliferation, contributing to growth and development in the body. Proper Fe homeostasis is essential for mitochondrial functioning, cellular respiration, and the subsequent production of ATP (the energy currency of cells). It is also a part of several proteins involved in the electron transport chain, which is critical for generating energy in the form of ATP during cellular respiration. Within the central nervous system (CNS), Fe is the most abundant trace element and is involved in a wide range of processes. It plays a role in the synthesis of neurotransmitters like dopamine and serotonin, which are crucial for brain function. Fe also influences synaptic plasticity (the ability of synapses to change strength) and myelination (the formation of myelin sheaths around nerve fibers for better nerve impulse conduction). Proper Fe concentrations in the brain are regulated by ferritin, a protein that stores excess iron. Balanced Fe homeostasis and concentrations are vital for maintaining proper cognitive functions and supporting neurodevelopmental processes. In aging individuals, Fe accumulation within the brain might occur and could be associated with cognitive and motor dysfunctions. Imbalances in Fe levels, either due to deficiency or overload, can result in impaired monoamine neurotransmission (communication between nerve cells that use neurotransmitters like dopamine, serotonin, etc.) or cellular toxicity with potential neuronal damage, respectively.	[38,39,40,41,42]
Zinc (Zn)	70–125 µg/dL (serum)		Zinc (Zn) is the second most abundant trace element in the human body. It plays crucial roles in various physiological processes, including the maintenance of protein structure, regulation of gene expression, and RNA and DNA synthesis. As a result, Zn is essential for proper cell development, replication, and metabolism. Zinc serves as a cofactor for numerous enzymes, including dopamine β-hydroxylase, monoamine oxidase, tyrosinase, alkaline phosphatase, carbonic anhydrase, superoxide dismutase, DNA and RNA polymerases, alcohol dehydrogenase, and matrix metalloproteinases. In the CNS, zinc is most abundant in the hippocampus and the olfactory bulb, mainly found in the synaptic vesicles of glutaminergic neurons. Zn-containing neurons are highly concentrated in the forebrain. Zinc can inhibit the release of glutamate (Glu) and affect γ-aminobutyric acid type A (GABAA) receptors, which play a role in neurotransmission. Proper levels of zinc are crucial for adult neurogenesis and proper hippocampal functioning. Altered zinc levels within the CNS can lead to various neurological disorders, including cognitive impairments, mood disorders, anxiety, depression, epilepsy, Alzheimer’s disease, and dementia. Zinc is also implicated in neuronal damage. Symptoms of zinc deficiency include growth retardation, mental lethargy, alterations in hormone metabolism, impaired immunity, and cognitive dysfunctions. Additionally, zinc deficiency can contribute to the promotion of inflammation in the body.	[43,44,45,46,47,48]
Magnesium (Mg)	0.65–1.05 mmol/L (total Mg in serum) 0.55–0.75 mmol/L (ionized Mg in serum)		Approximately 99% of the total body magnesium (Mg) is found in bones and muscles. Magnesium serves as a cofactor for more than 300 enzymes, which are involved in a wide range of functions within the body. These functions include neuromuscular conduction, muscle contraction, myocardial contraction (heart muscle function), and regulation of blood pressure. Mg is required for glycolysis (the breakdown of glucose), energy production, and oxidative phosphorylation (a process in cellular respiration). It also plays a role in controlling N-methyl-D-aspartate (NMDA) receptors in the brain, helping to prevent neuronal overstimulation. As a crucial mineral for bone health, magnesium is essential for proper bone mineralization. It is also necessary for maintaining the structures of proteins, nucleic acids (DNA and RNA), and mitochondria (the energy-producing organelles in cells). Additionally, magnesium is involved in the proper transmembrane transport of ions across cell membranes. Magnesium plays a role in several immunological functions, such as macrophage activation and lymphocyte proliferation, contributing to a well-functioning immune system. A magnesium deficiency can affect multiple systems in the body, leading to various symptoms. Neurologically, magnesium deficiency is associated with a higher risk of migraines, strokes, and seizures. Gastrointestinal symptoms may include insulin resistance, increased levels of triglycerides, and total cholesterol. Cardiovascular symptoms may include an increased risk of hypertension (high blood pressure) and atherosclerosis (hardening and narrowing of arteries), and patients with magnesium deficiency also present a higher risk of osteoporosis. On the other hand, excessive levels of magnesium (hypermagnesemia) can lead to symptoms such as hypotension (low blood pressure), nausea, vomiting, and cutaneous flushing. More severe cases of hypermagnesemia may cause neuromuscular dysfunctions, bradycardia (slow heart rate), atrial fibrillation (irregular heart rhythm), respiratory depression, or even coma.	[49,50,51,52,53]
Copper (Cu)	70–140 mcg/dL (blood)		Copper (Cu) is the third most common transition element in the human body, and its highest concentrations are found primarily in the liver and brain. Copper plays diverse and essential roles in various physiological processes. One of the significant functions of copper is its involvement in proper iron (Fe) homeostasis, ensuring the balance of iron levels in the body. Copper is also crucial for myelination, the process by which nerve fibers are coated with a protective sheath, supporting efficient nerve signal transmission. Additionally, copper is essential for neurotransmitter synthesis, including the production of dopamine and norepinephrine, which are important for brain function and mood regulation. Copper is a vital component of several enzymes, such as tyrosine hydroxylase and dopamine hydroxylase, which are involved in the production of neurotransmitters. Other enzymes include superoxide dismutase, an antioxidant enzyme, and cytochrome c oxidase, which plays a role in cellular respiration. Approximately 80–95% of copper in the plasma is bound to ceruloplasmin, a copper-binding protein that helps transport copper in the blood. Copper is also stored in metallothionein, an important protein that aids in copper storage. However, excessive copper can be toxic, mainly due to oxidative damage caused by free radicals. Copper can bind to GABAA, NMDA receptors, and voltage-gated Ca2+ channels, impairing synaptic transmission. Copper concentrations and metabolic imbalances have been implicated in various neurodegenerative diseases, such as Alzheimer’s disease, Menkes disease (a genetic disorder affecting copper metabolism), Wilson’s disease (a genetic disorder causing copper accumulation), and spongiform encephalopathy (a group of neurological disorders involving abnormal protein folding).	[43,54,55,56,57,58,59]
Aluminium (Al)	<10 µg/L (serum)		Patients exposed to elevated levels of aluminum (Al) experience an accumulation of this metal in both their blood plasma and brain. The entry of Al into the CNS is facilitated through transferrin, concentrating mainly in regions rich in transferrin receptors. When plasma Al levels exceed 13 µg/L, early symptoms of neurotoxicity may become evident. Additionally, excessive Al exposure can trigger inflammatory responses by upregulating the expression of NF-κB and TNF-α. Accumulation of Al in the brain has been associated with several cognitive dysfunctions and, in more advanced stages, even dementia. Furthermore, this metal can disrupt hippocampal calcium (Ca) signaling pathways. The neurotoxic effects of Al are closely related to oxidative stress and the impaired synthesis of acetylcholine, particularly affecting cholinergic neurons, which are susceptible to Al toxicity. Moreover, elevated Al levels can also negatively impact acetylcholinesterase (AChE) activity and impair the functions of glial cells. Long-term exposure to Al can lead to conditions such as aluminosis, encephalopathy, breast cancer, and Alzheimer’s disease. As time passes, the physiological content of Al tends to increase within the brain due to these harmful processes. It is crucial to monitor and regulate Al exposure to mitigate its potential detrimental effects on neurological health.	[60,61,62,63,64]
Cadmium (Cd)	0.5–2.0 ng/mL (blood)		Cadmium (Cd) is a toxic heavy metal with no significant biological function in the human body. It has been classified as a human carcinogen due to its ability to disrupt DNA repair, leading to uncontrolled cellular proliferation. Additionally, Cd facilitates the overexpression of various proto-oncogenes like c-myc or c-jun, further contributing to cancer risk. The metal also induces oxidative stress. Currently, Cd contamination is widespread in many food products, and its accumulation in the human body tends to increase with age. Cd is commonly found to accumulate in the liver, lungs, and eye tissues. Chronic exposure to Cd has been linked to diseases such as Itai-itai disease, tubular impairments associated with bone demineralization, and osteoporosis. Furthermore, there is evidence suggesting an association between Cd levels and the risk of diabetes, diabetic nephropathy, hypertension, and periodontal diseases. As a carcinogen, chronic exposure to Cd may lead to tumorigenesis in various organs, including the lungs, pancreas, prostate, stomach, and bladder. Moreover, prolonged exposure to Cd may result in neuropsychological dysfunctions, including cognitive delay.	[65,66]
Calcium (Ca)	8.6–10.2 mg/dL (blood)		Calcium (Ca) is the most abundant mineral in the human body, with approximately 99% found in bones and only 1% in the serum. Ca metabolism is closely linked to several essential nutrients, among which phosphorus (P) and vitamin D play a major role. This mineral plays a crucial role in various physiological processes, including proper nerve transmission, vasoconstriction with vasodilation, muscle contraction, and intercellular signaling. Maintaining the appropriate Ca2+ homeostasis is achieved through two types of Ca2+ transport ATPases: the plasma membrane Ca2+-ATPase (PMCA) and the intracellular sarco/endoplasmic reticulum Ca2+-ATPase. Neuronal and glial cells express Ca-sensing receptors that are activated by extracellular Ca. These receptors play a role in neurotransmission and synaptic plasticity. Furthermore, Ca ions are involved in initiating and regulating responses to injuries within the CNS. Astrocytic Ca signals can modulate synaptic transmission, and proper glial cell function is also influenced by Ca signaling. Ca overload can lead to Glu excitotoxicity, stroke, and various neurodegenerative diseases. Conversely, Ca deficiency may result in calcification of the cerebellum, cerebral cortex, and basal ganglia, leading to subsequent extrapyramidal signs. Other effects of Ca deficiency include irritability, increased intracranial pressure, and spasms. On the other hand, hypercalcemia, which is an excessive level of Ca in the blood, primarily affects endocrine function due to increased parathormone production.	[67,68,69,70]
Manganese (Mn)	0.4–0.85 µg/L (serum)		Manganese (Mn) is an essential element crucially involved in regulating glucose and lipid metabolism, as well as the synthesis and activation of various enzymes. These enzymes include arginase, isocitrate dehydrogenase, phosphoenolpyruvate carboxykinase, manganese superoxide dismutase (MnSOD), glutamine synthetase, glycosyl transferases, and pyruvate carboxylase. As a result, Mn plays a significant role in proper development, antioxidant defense, energy production, immune responses, and neuronal activity. The CNS has the highest concentrations of manganese, predominantly found in regions like the putamen, caudate nucleus, and globus pallidus. However, molecular mechanisms associated with Mn toxicity are numerous and include oxidative stress, mitochondrial dysfunction, autophagy dysregulation, and apoptosis, among others. Manganese toxicity disrupts the glutamine (Gln)/Glu-gamma-aminobutyric acid (GABA) cycle between astrocytes and neurons, impairing neurotransmission and Gln metabolism. Such imbalances in Mn levels are linked to neurodegenerative diseases. Excessive Mn levels, a condition known as manganism, can present symptoms similar to Parkinson’s disease, including cognitive, motor, and emotional impairments, and may even induce encephalopathy. Excessive amounts of Mn in the body are neurotoxic, and this mechanism is further enhanced by Mn-related overactivation of glial cells, leading to neuroinflammatory responses. Proper regulation of Mn levels is crucial for maintaining healthy neurological function and preventing neurotoxicity and associated disorders.	[43,71,72,73,74]
Nickel (Ni)	0.2 µg/L (serum)		Nickel (Ni) is highly abundant in nucleic acids, particularly in RNA. It serves as a crucial component of several enzymes, including glyoxalase I, acireductone dioxygenase, nickel superoxide dismutase, ureases, Ni-Fe hydrogenase, methyl-CoM reductase, and CO dehydrogenase. These enzymes play essential roles in various metabolic processes. Ni is involved in important physiological functions, such as iron absorption and erythrocyte synthesis, as well as the metabolism of adrenaline, glucose, hormones, lipids, and cell membranes. However, excessive exposure to nickel can lead to various side effects and health issues. Commonly reported adverse effects of excessive Ni exposure include lung fibrosis, skin allergies, and an increased risk of developing nasal, laryngeal, and lung cancers. The Ni toxicity syndrome’ is characterized by a wide range of symptoms, including hypoglycemia, shortness of breath, nausea, a lowered pulse rate, headaches, diarrhea, and vomiting. Severe nickel intoxication can significantly impact the respiratory tract and gastrointestinal system. The most common causes of death resulting from Ni intoxication are pneumonitis (inflammation of the lungs) and cerebral edema (fluid accumulation in the brain).	[75,76,77,78,79]
Molybdenum (Mo)	0.28–1.17 ng/mL (serum)		Molybdenum (Mo) serves as a vital cofactor for three main enzymes in the body: Sulphite oxidase: This enzyme is involved in the metabolism of sulfur-containing amino acids. Xanthine oxidase/dehydrogenase: It catalyzes the oxidative hydroxylation of purines and pyridines. Aldehyde oxidase: This enzyme is responsible for oxidizing purines, pyrimidines, and pteridines. A deficiency of sulphite oxidase can lead to neurological symptoms. A low dietary intake of molybdenum results in decreased concentrations of serum and urinary uric acid, as well as excessive excretion of xanthine. Since molybdenum is required in small amounts, its deficiency is relatively rare. However, in cases of “acquired Mo deficiency,” characterized by high blood methionine levels, low blood uric acid levels, and reduced urinary sulfate and uric acid levels, motor dysfunctions might be associated.	[80,81,82,83,84,85]
Phosphorus (P)	2.5 to 4.5 mg/dL (blood)		Phosphorus (P) is an essential element involved in numerous vital processes in the body. It plays a fundamental role in DNA and ATP synthesis, membrane formation, and protein phosphorylation. P is a critical component of DNA and RNA, which are essential for genetic information and cellular functions. Inorganic phosphate is particularly important for proper skeletal mineralization, with approximately 85% of phosphorus distributed within bones and teeth, while the remaining quantities are found in blood and other tissues. Phosphorus plays a key role, either directly or indirectly, in various biological processes such as gene transcription regulation, cell signaling through phosphorylation reactions, maintaining acid-base homeostasis to ensure the physiological pH of bodily fluids, activating numerous enzymes, and proper energy storage. It is also a component of 2,3-diphosphoglycerate, which plays a role in oxygen transport in red blood cells. Phosphorus deficiency can lead to numerous bone-related symptoms, such as increased bone pain, fragility, and joint stiffness. Muscle dysfunctions, primarily affecting major muscles, are also common, with severe cases potentially leading to respiratory depression and low cardiac output. Chronic phosphorus deficiency can result in proximal myopathy, rhabdomyolysis (an increased risk of hemolytic anemia), and impaired erythrocyte synthesis. Hypophosphatemia, characterized by low levels of phosphorus in the blood, can also lead to neurological or cognitive symptoms, including fatigue, weakness, irritability, apathy, intention tremors, delirium, or even coma. On the other hand, hyperphosphatemia, characterized by elevated levels of phosphorus in the blood, can manifest as enhanced vascular or soft tissue calcification, an increased risk of secondary hyperparathyroidism, and renal osteodystrophy.	[86,87,88]
Uranium (U)			Uranium (U) is a heavy metal that can be absorbed into the human body through various routes, including inhalation, ingestion of U-contaminated food and water, and dermal contact (e.g., through damaged tissues). U toxicity can manifest as either acute or chronic, and it primarily affects the kidneys, with other organs such as bones, liver, lungs, and reproductive organs also susceptible to chronic toxicity. Uranium can pass through the blood-brain barrier, leading to concerns about its potential neurotoxic effects. So far, U has shown toxic properties, particularly toward dopaminergic cells in the brain. Chronic exposure to uranium can also impact the immune system, resulting in a wide spectrum of effects ranging from infectious diseases to autoimmune disorders. Additionally, there are concerns about uranium’s potential to induce carcinogenesis or the development of cancer.	[89,90,91,92,93]
Chromium (Cr)	0.5 and 2.5 μg/L (blood) 0.8 and 5.1 μg/L (serum)		Chromium (Cr) is a vital trace element necessary for normal carbohydrate metabolism. Its biological function is closely linked to insulin, and many reactions stimulated by chromium are also dependent on insulin. A deficiency of chromium (III) can lead to disturbances in metabolic processes. One of the primary responses of the body to chromium (III) deficiency is a decreased glucose tolerance, resulting from changes in insulin’s affinity to its receptors on cells. Additionally, significant amounts of chromium (III) are found in nucleic acids, which influences their metabolism, replication, and transcription processes. Furthermore, chromium ions can reduce the levels of corticosteroids in the plasma and enhance the functional activity of the immune system in the body. In summary, chromium is essential for proper carbohydrate metabolism and is closely associated with insulin function. A chromium deficiency (III) can lead to metabolic disruptions, altered glucose tolerance, and impacts on nucleic acid metabolism and immune system activity.	[94,95,96]
Lead (Pb)			Lead (Pb) exposure can have severe and harmful effects on different systems in the body, including the hematopoietic (blood-forming), renal (kidney), reproductive, and central nervous systems. The primary mechanism of these deleterious effects is the increase in oxidative stress, which leads to cellular damage and dysfunction. It is essential to note that there is no known level of lead that is necessary or beneficial for the body. Even at low levels, lead exposure can be harmful. Consequently, no “safe” level of exposure to lead has been identified. Any level of lead exposure can potentially lead to adverse health effects, especially over prolonged periods.	[97]
Mercury (Hg)			Human toxicity caused by mercury (Hg) can vary based on the specific form of mercury, the dose, and the rate of exposure. Different forms of mercury have distinct effects on the body. When mercury vapor is inhaled, the primary target organ is the brain. Mercurous and mercuric salts, on the other hand, tend to damage the gut lining and the kidneys. Methyl mercury, a form of organic mercury, is distributed widely throughout the body. The severity of toxicity depends on the dosage. Large acute exposures to elemental mercury vapor can lead to severe pneumonitis, a condition characterized by inflammation of the lungs, which, in extreme cases, can be fatal.	[98]
Arsenic (As)			Arsenic (As) acts as a potent poison that disrupts cellular functions by targeting sulfhydryl groups within cells. It interferes with crucial cell processes such as enzymatic activity, cell respiration, and cell division (mitosis). Two significant forms of arsenic, inorganic arsenite (III) and organic arsenicals with the general formula R-As2+ form strong bonds with thiol groups, particularly with vicinal dithiols like dihydrolipoic acid (DHLA). These thiol groups, along with certain seleno-enzymes, become vulnerable targets for the toxic effects of arsenic. Furthermore, R-As2+-compounds exhibit an even higher affinity for selenol groups, found in proteins like thioredoxin reductase, which also contain a thiol group adjacent to the selenol. The inhibition of these reactive oxygen species (ROS)-scavenging seleno-enzymes is responsible for the oxidative stress associated with arsenic poisoning. Overall, arsenic’s ability to disrupt cellular functions by targeting sulfhydryl and selenol groups in critical enzymes and proteins leads to oxidative stress, contributing to the harmful effects of arsenic poisoning on the body.	[99,100]
Antimony (Sb)			Exposure to high concentrations of antimony in the air, specifically at levels of 9 mg/m^3^, can lead to irritation of the eyes, skin, and lungs. Prolonged exposure to antimony in smelting plants, in particular, may result in the development of antimoniosis, a specific type of pneumoconiosis that affects the lungs. Chronic exposure to antimony is associated with an increased risk of lung, heart, and gastrointestinal diseases. The health effects of exposure to antimony can include vomiting and irritation of the eyes and mucous membranes. However, antimony compounds are generally not considered to pose significant mutagenic, carcinogenic, or teratogenic risks in pregnant women. This means that, in general, antimony exposure is not believed to cause genetic mutations, cancer, or birth defects in pregnant women or their unborn children.	[101]

## Data Availability

Not applicable.
